# La détresse psychologique du personnel médical et paramédical d’anesthésie-réanimation

**DOI:** 10.11604/pamj.2018.29.221.12189

**Published:** 2018-04-23

**Authors:** Najla Halouani, Mariem Turki, Rihab Ennaoui, Jihène Aloulou, Othman Amami

**Affiliations:** 1 Service de Psychiatrie “B”, CHU Hédi Chaker, Sfax, Tunisie

**Keywords:** Stress, anxiété, anesthésie, Stress, anxiety, anesthesia

## Abstract

L’anesthésie-réanimation (AR) est réputée être l’une des spécialités les plus touchées par le stress professionnel (SP). Dans ce contexte, notre travail avait pour objectifs d’évaluer le niveau d’anxiété et de SP chez le personnel d’AR et d’identifier les facteurs qui y sont associés. Pour ce faire, nous avons mené une étude transversale, descriptive et analytique, auprès de 54 participants (38 techniciens et 16 résidents) exerçant aux deux services d’AR des CHU Habib Bourguiba et Hédi Chaker de Sfax, Tunisie. L’évaluation du SP a été faite via le questionnaire de Karasek. L’échelle « Hamilton Anxiety Rate Scale » (HARS) a été utilisée pour la mesure de l’anxiété. Selon le questionnaire de Karasek, le score moyen de la latitude décisionnelle était de 69 points, celui de la demande psychologique 23,9 points, celui du soutien social 19,6 points. Selon ce questionnaire, 40,7% du personnel étaient estimés stressés et 38,9% en situation d’isostrain. Le score HARS moyen était de 17,8 points, celui de l’anxiété psychique 8,7 points et celui de l’anxiété somatique 9 points, avec une différence significative entre le personnel médical et paramédical. Une anxiété sévère à très sévère a été notée dans 25,9% des cas. Le score HARS était corrélé au sexe féminin (p = 0,017) et aux antécédents de suivi psychiatrique (p = 0,003). Il ressort de notre étude que le personnel d’AR est exposé à un risque considérable de SP. Des changements de l’environnement de travail ainsi qu’un apprentissage de techniques de gestion du stress professionnel pourraient être proposés.

## Abstract (en)

Anesthesia and intensive care medicine is considered one of the specialties involving more professional stress (PS). On this basis, our study aimed to assess the level of anxiety and PS in the personnel in anesthesia and intensive care and to identify the associated factors. We conducted a cross-sectional, descriptive and analytical study including 54 participants (38 technicians and 16 residents) working in the Departments of Anesthesia and Intensive Care at Habib Bourguiba and Hedi Chaker University Hospitals, Sfax, Tunisia. PS assessment was performed using the Karasek questionnaire. The Hamilton Anxiety Rating Scale (HARS) was used to measure the severity of anxiety symptoms. According to the Karasek questionnaire, the average decision latitude score was 69, the average psychological demand score was 23.9 and the average social support score was 19.6. According to this questionnaire, 40.7% of personnel was stressed and 38.9% was in isostrain condition. Average HARS score was 17.8, average psychic anxiety score was 8.7 and average somatic anxiety score was 9, with a significant difference between the medical and paramedical personnel. Severe to very severe anxiety was recorded in 25.9% of cases. HARS score was correlated to female gender (p=0.017) and to psychiatric histories (p=0.003). This study highlights that medical and paramedical personnel in anesthesia and intensive care is exposed to a significant risk of PS. Changes in working environment as well as learning professional stress management techniques should be proposed.

## Introduction

L’évaluation des risques psychosociaux dans le monde du travail et plus particulièrement du stress est au cœur des préoccupations actuelles [1]. Après avoir été longtemps sous-estimé voire même méprisé, le stress engendré par le travail et ses conséquences font aujourd’hui l’objet d’une attention particulière dans les professions exposées, dont les professions médicales [2]. L’anesthésie-réanimation (AR) est réputée être l’une des spécialités les plus touchées par le stress professionnel. Elle s’attache à prendre en charge les patients présentant des pathologies graves, susceptibles de présenter une ou plusieurs défaillances viscérales aiguës mettant directement en jeu le pronostic vital [2]. Le personnel médical et paramédical est souvent confronté à des situations graves et imprévisibles, qui sont génératrices d’une anxiété et d’un stress intenses et difficiles à maitriser. Dans ce contexte, notre travail avait pour objectifs d’évaluer le niveau d’anxiété et de stress professionnel chez le personnel d’AR et d’identifier les facteurs qui y sont associés.

## Méthodes

Nous avons mené une étude observationnelle, transversale, descriptive et analytique réalisée pendant le mois de septembre 2014 auprès de 54 participants (38 techniciens et 16 résidents) exerçant aux deux services d’AR des CHU Habib Bourguiba et Hédi Chaker de Sfax. Le recueil des données a été effectué à l’aide d’une fiche pré-établie précisant les caractéristiques sociodémographiques et cliniques.

L’évaluation du stress professionnel a été faite via le questionnaire de Karasek. Il s’agit d’un outil à 29 items pour l’évaluation des facteurs psychosociaux et du bien-être au travail. Trois dimensions sont ainsi mesurées: 1) la latitude décisionnelle (qui comporte 2 sous-dimensions: l’utilisation des compétences qui se définit par la possibilité d’utiliser et développer ses compétences et qualification et l’autonomie décisionnelle qui se définit par la marge de manœuvre dans la manière de faire son travail et de prendre part aux décisions qui s’y rattachent); 2) la demande psychologique, qui porte sur des aspects aussi bien quantitatifs que qualitatifs de la charge psychologique de travail; 3) et le soutien social, qui comporte des aspects relatifs au soutien socio-émotionnel et instrumental des relations avec la hiérarchie et les collègues.

**Figure 1:**
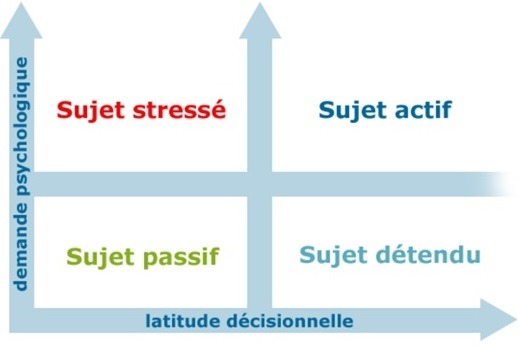
Le modèle de Karasek

Le modèle de Karasek [3] permet ainsi de situer les travailleurs sur un graphique défini selon deux axes: la demande psychologique (seuil = 21) et la latitude décisionnelle (seuil = 70) (Figure 1). Un sujet stressé, ou en en situation de « job strain », dispose ainsi d’une forte demande psychologique et d’une faible latitude décisionnelle (donc une faible autonomie). La situation d’ « iso strain » est définie par l’association d’un « job strain » et d’un faible soutien social (seuil = 24). D’un autre côté, l’échelle de « Hamilton Anxiety Rate Scale » (HARS) a été utilisée pour la mesure de l’anxiété [4]. Il s’agit d’une échelle à 14 items, cotés chacun de 0 à 4. Sept items explorent l’humeur anxieuse, et sept autres items évaluent les symptômes somatiques associés à l’anxiété.

L’anxiété est légère si le score est ≤ 17; modérée si le score est compris entre 18 et 24; sévère pour un score 25 - 30 et très sévère pour un score ≥ 30. L’analyse statistique était effectuée à l’aide du logiciel SPSS dans sa version 20. Les différences entre les résultats ont été considérées significatives pour une valeur de p < 0,05.

## Résultats

**Données socio-démographiques:** L’’âge moyen des enquêtés était de 41,68 ans (min: 27; max: 59). Le sex-ratio F/H était de 2,6 (il s’agissait de 15 hommes et 39 femmes). Parmi les participants, 83,3% étaient mariés. Des antécédents médicaux et chirurgicaux étaient notés respectivement dans 29,6% et 31,5% des cas. Treize pour cent des participants avaient rapporté avoir un suivi en psychiatrie.

**Les données relatives au travail:** Le nombre moyen d’années de travail était de 16,20 ans (min 1, max 34 ans). Le travail posté était rapporté dans 63% des cas. Le Tableau 1 résume les principaux facteurs subjectifs jugés comme générateurs de stress chez le personnel d’AR.

**Tableau 1:** Facteurs perçus comme générateurs de stress

		**Personnel médical**	**Personnel paramédical**	**Tout le personnel**	**p**
Facteurs liés à la tâche à effectuer	exigences importantes (surcharge de travail)	50%	84,2%	74,1%	**0,009**
	difficultés liées à la tâche elle-même	6,2%	52,6%	38,9%	**0,002**
	risques inhérents à l'exécution même de la tâche	31,2%	42,1%	38,9%	NS
	Peur d’erreur médicale	25%	52,6%	44,4%	NS
Facteurs liés à l'organisation du travail	L'absence de contrôle sur la répartition et la planification des horaires parfois incompatibles avec une vie familiale et sociale	75%	44,7%	53,7%	NS
	Absence de récupération post-garde	93,8%	10,5%	35,2%	**<0,001**
	Effectif réduit du personnel	12,5%	94,7%	70,4%	**<0,001**
	Pressions administratives	6,2%	15,8%	13%	NS
Facteurs liés aux relations de travail	manque d'aide de la part des collègues	0%	26,3%	18,5%	**0,024**
	manque d'aide de la part des supérieurs hiérarchiques	87,5%	63,2%	70,4%	NS
Facteurs liés à l'environnement physique et technique	Nuisances physiques au travail (bruit, chaleur, humidité, poussière…)	25%	92,1%	72,2%	**<0 ,001**
	Mauvaise conception des lieux et/ou des postes de travail (manque d'espace, éclairage inadapté…)	43,8%	60,2%	55,6%	NS
	Manque d'hygiène	75%	71,1%	72,2%	NS
Déséquilibre entre vie privée et vie professionnelle		62,5%	18,4	31,5%	**0,001**
Rapport salaire/effort insatisfaisant		87,5%	61,2%	70,4%	NS

NS: Non Significatif; %: effectif

**Figure 2:**
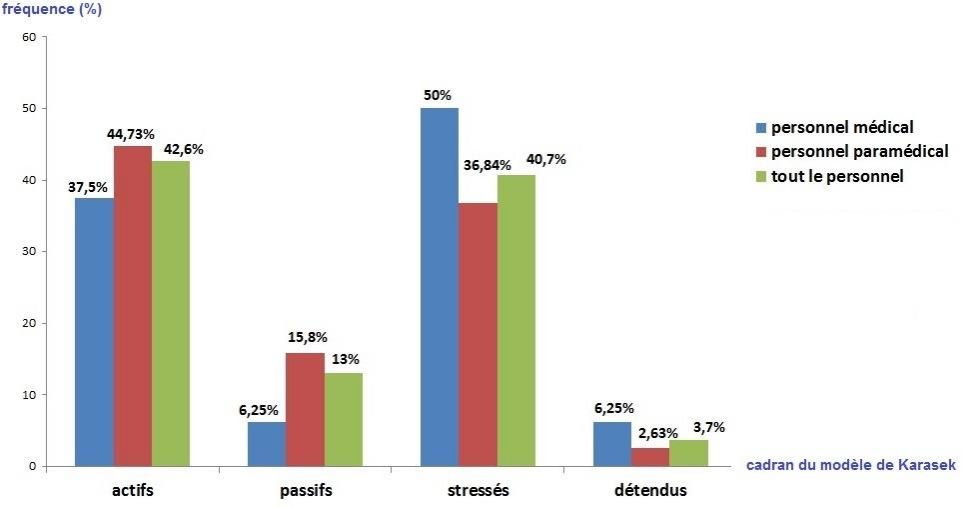
Répartition du personnel selon le modèle de Karasek

**Le score de Karasek:** Le score moyen de la latitude décisionnelle était de 69 points, soit en faveur d’une faible latitude, sans différence significative entre le personnel médical et paramédical. Le score moyen de la demande psychologique était de 23,9 points, soit en faveur d’une forte demande. De même, il n’y avait pas de différence significative entre les deux groupes. Le score moyen du soutien social était de 19,6 points, soit en faveur d’un faible soutien. Il était significativement plus élevé chez le personnel paramédical (20,47 vs 17,62; p = 0,016). Sur le Tableau 2 figure une comparaison entre le personnel médical et paramédical concernant les différentes dimensions du questionnaire de Karasek. Selon ce modèle, 40,7% des participants étaient estimés stressés ou en situation de jobstrain, 42,6% actifs, 13% passifs et 3,7% détendus (Figure 2). Une situation d’isostrain a été notée chez 38,9% du personnel (Tableau 2). La Figure 2 illustre la répartition du personnel médical et paramédical selon les quadrants du modèle de Karasek.

**Tableau 2:** Dimensions du score de Karasek chez le personnel médical et paramédical

Dimension		Personnel médical	Personnel paramédical	Tout le personnel	p
Latitude décisionnelle	faible	56,2%	52,6%	53,7%	NS
	forte	43,8%	47,4%	46,3%	
Demande psychologique	faible	12,5%	18,4%	16,7%	NS
	forte	87,5%	81,6%	83,3%	
Soutien social	faible	100%	76,3%	83,3%	**0,045**
	fort	0%	23,7%	16,7%	
jobstrain	oui	50%	36,8%	40,7%	NS
	non	50%	63,2%	59,3%	
isostrain	oui	50%	34,2%	38,9%	NS
	non	50%	65,8%	61,1%	

NS :Non Significatif; % :effectif

**Figure 3:**
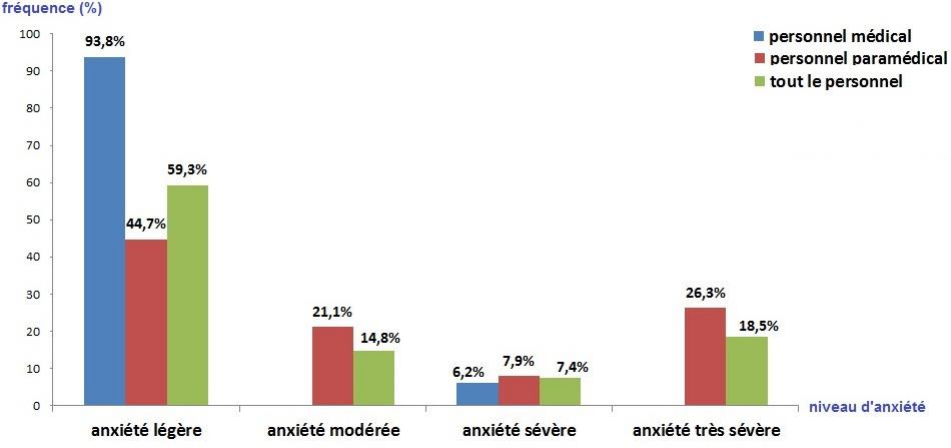
Niveau d’anxiété chez le personnel d’anesthésie

**Le score d’Hamilton:** Le score HARS moyen était de 17,8 points, celui de l’anxiété psychique: 8,7 points et celui de l’anxiété somatique 9 points. Une anxiété sévère à très sévère a été notée chez 25,9% du personnel (Figure 3). Le Tableau 3 et la Figure 3 illustrent la différence entre l’équipe médicale et paramédicale selon le niveau d’anxiété.

**Tableau 3:** Scores d’anxiété selon l’échelle HARS

	équipe	Score (points)	p
Score total moyen	personnel médical	9,5	**<0,001**
	personnel paramédical	21,3	
Score moyen d’anxiété psychique	personnel médical	5,87	**0,003**
	personnel paramédical	9,9	
Score moyen d’anxiété somatique	personnel médical	3,6	**< 0,001**
	personnel paramédical	11,34	

**Les facteurs corrélés à l’anxiété et au stress professionnel:** La situation de « stress » ou « tension au travail » ou « jobstrain » était plus fréquente chez le personnel médical (les résidents), les femmes, les jeunes âgés de moins de 40 ans, en cas de travail posté, de faible nombre d’années de travail< 10 ans, et de suivi pour une pathologie somatique, sans que la relation soit statistiquement significative. Le score HARS était corrélé au sexe féminin (p = 0,017), ainsi qu’aux antécédents de suivi psychiatrique (p = 0,003).

## Discussion

### Le stress professionnel

Le stress est une problématique préoccupante du monde du travail. Selon l’agence européenne pour la sécurité et la santé au travail, le stress professionnel survient «lorsqu’il y a un déséquilibre entre la perception qu’une personne a des contraintes que lui impose son environnement et ses propres ressources pour y faire face». Plusieurs modèles ont été utilisés dans la mesure du stress au travail, dont les plus documentés sont le modèle du « déséquilibre efforts/récompenses » de Siegrist [5] et le modèle « demande-autonomie » de Karasek [3]. Le premier s’appuie sur l’hypothèse qu’une situation de travail caractérisée par une combinaison d’efforts élevés et de faibles récompenses est suivie de réactions pathologiques sur le plan émotionnel et physiologique. Selon le deuxième, un niveau élevé de demandes au travail associé à un niveau faible de latitude de décision aboutit à un niveau élevé de tension. Ce modèle a été enrichi en 1990 par l’introduction d’un facteur supplémentaire, le soutien social. Ce dernier confirme l’incidence de variables psychosociales dans l’évaluation de l’intensité de stress perçu [6]. C’était justement la complémentarité cette approche tridimensionnelle qui nous a incité à utiliser ce modèle pour la mesure du stress professionnel dans notre étude. Selon ce modèle, nos participants se sont situés dans le cadran « actif» dans 42,6% des cas, « stressé » dans 40,7% des cas, « passif» dans 13% des cas et estimés « détendus » dans 3,7% des cas. Nos résultats vont de pair avec la théorie de Karasek. En effet, selon Karasek [6], le cadran « actif », où les exigences et l’autonomie sont élevées, inclut les professions présumées prestigieuses : avocats, juges, médecins, professeurs, ingénieurs, personnel infirmier et cadres de toutes sortes. Cependant, une hétérogénéité a été notée entre le personnel médical et paramédical: les médecins ont été estimés en majorité « stressés », donc ayant une plus faible autonomie. La situation de stress a été notée chez la moitié des médecins et plus qu’un tiers (37,8%) des techniciens d’anesthésie.

Dans la littérature, peu d’études ont examiné sur des critères objectifs le stress chez les anesthésistes [7]. Malmberg et al. [8] ont observé une modification significative de la TSH après une nuit de garde chez les anesthésistes. Le syndrome d’épuisement professionnel, un stade ultime du stress professionnel, semble être fréquent chez l’ensemble du personnel médical et paramédical en anesthésie réanimation. Il toucherait ainsi entre 25 à 50 % des médecins selon les études [9] et entre 20 et 40 % du personnel paramédical [10]. Plusieurs facteurs ont été incriminés dans la genèse du stress. Pour les participants paramédicaux, les exigences importantes du travail, la peur d’erreur médicale, l’effectif réduit du personnel, les nuisances physiques au travail, et le manque d'hygiène étaient les facteurs les plus rapportés.

Quant aux médecins résidents, l’absence de récupération post-garde, le manque d'aide de la part des supérieurs hiérarchiques, le manque d'hygiène, le déséquilibre entre la vie privée et la vie professionnelle et le rapport salaire/effort insatisfaisant étaient les facteurs les plus incriminés. Nos résultats rejoignent les données de la littérature. Selon une enquête marocaine [11], réalisée en 2009 aux services d’anesthésie-réanimation, l’organisation défaillante des services, la crainte de l’erreur médicale et le salaire non satisfaisant étaient associés à un risque relatif d’épuisement professionnel respectivement de 1,88; 2,09 et 1,93. Selon une autre étude [12], les facteurs qui contribuent au stress des anesthésistes sont la proximité de la souffrance et la mort, les besoins physiques et émotionnels des patients et la pression d’obtenir de bons résultats. Le facteur rapport salaire/effort insatisfaisant est rarement mentionné dans les pays développés [11], mais il parait compréhensible de le retrouver dans notre contexte où la récompense financière du soignant (surtout les jeunes résidents en formation) reste très modeste par rapport à l’effort fourni.

Bien que la relation ne fût pas statistiquement significative, nous avons noté que la situation de stress était plus fréquente chez les résidents, les plus jeunes, ayant un moindre nombre d’années de travail. Ceci pourrait s’expliquer par le manque d’expérience professionnelle, qui implique un double effort d’adaptation inhérent à l’incertitude du débutant quant à ses compétences et à la confrontation à des situations nouvelles où l’imprévisibilité prend une dimension encore plus importante [13]. D’ailleurs, selon certains auteurs, l’épuisement professionnel diminuerait avec l’ancienneté professionnelle [14]. Il y aurait un pic d’incidence en début de carrière, dans les cinq premières années d’activité pour la plupart des études [15,16]. Le rôle protecteur de l’expérience professionnelle a été retrouvé dans de nombreuses études [14,17]. Il concerne essentiellement des médecins résidents (moins de cinq ans d’activité). Cela peut être expliqué par plusieurs facteurs dont le manque d’encadrement, un sentiment d’abandon face aux situations difficiles, une surcharge de travail, un manque de sommeil et de temps libre [14,18,19]. La surcharge de travail est fréquemment associée au burnout dans les différentes études [9,20]. En particulier, un des facteurs de risque principal semble être le nombre de garde par mois [9].

Dans notre étude, les participants ayant un travail posté sont plus exposés au stress, sans que la relation soit significative. Selon Magalhães et al. [12], le manque de sommeil et la fatigue résultant du travail de nuit contribuent à un déficit de l'attention, perturbation des fonctions cognitives et des réflexes. Par ailleurs, nous avons rapporté une plus grande fréquence de stress professionnel chez les femmes. Ceci pourrait être expliqué par des possibilités plus limitées de communication et de contrôle de leur travail par rapport aux collègues masculins. D’autre part, la double charge de travail et de la vie privée, telle qu'elle est vécue plus fréquemment par les femmes anesthésiologistes, peut-être la raison pourquoi les femmes, d'abord, semblent reconnaître des restrictions possibilités de contrôler le travail d'une manière plus sensible et, d'autre part, sont plus susceptibles de subordonner leurs intérêts personnels au travail dans le but d'économiser l'énergie [21].

### L’anxiété au travail

Dans le monde du travail, peu d’études ont évalué spécifiquement l’anxiété ou les troubles anxieux, pourtant si on se réfère à l’ensemble des travaux épidémiologiques, on peut penser que l’anxiété est souvent la première symptomatologie qui apparaît dans les troubles de l’adaptation, bien que ceux-ci aient été insuffisamment étudiés [22]. L’anxiété, si elle est la première conséquence du stress professionnel, a été peu étudiée comparativement à la dépression. Elle représente pourtant le véritable baromètre qui doit aider à identifier, autant sur le plan individuel que sur le plan collectif, les risques de développement de troubles psychiatriques [20]. Dans notre étude, une anxiété modérée à très sévère a été retrouvée chez 40,7% du personnel. Les scores de l’anxiété étaient significativement plus importants chez le personnel paramédical. On pourra expliquer ces résultats par des préoccupations extraprofessionnelles (la vie familiale, la charge des enfants, les problèmes de santé…) plus importantes chez les paramédicaux qui sont plus âgés que les résidents. Endler [23] explique qu’une importante conséquence de la perception ou de l’évaluation de situations menaçantes s’exprime par une augmentation transitoire de l’anxiété, plus précisément de l’anxiété-état. Il s’agit, en effet, de réactions émotionnelles qui se caractérisent par des sentiments de tension, de nervosité, d’inquiétude et par l’activation ou l’éveil du système autonome.

## Conclusion

Il ressort de notre étude que le personnel d’anesthésie-réanimation est exposé à un risque considérable de stress au travail, favorisé par certains facteurs comme l’âge jeune, le sexe féminin et le travail posté. Ceci pourrait avoir des conséquences néfastes tant sur le plan individuel que collectif et donc sur le rendement au travail. Ainsi, une meilleure connaissance des différentes situations et facteurs générateurs de stress et d’anxiété s’avère nécessaire et une évaluation plus systématique mériterait d’être proposée afin de pouvoir mettre en place des mesures individuelles mais aussi organisationnelles permettant de réduire les conséquences humaines et économiques. Ainsi, des changements de l’environnement de travail, ainsi qu’un apprentissage de techniques personnelles de gestion du stress professionnel pourraient être proposés.

### Etat des connaissances actuelles sur le sujet

- Le personnel d’anesthésie-réanimation est souvent confronté à des situations génératrices d’une anxiété et d’un stress intenses et difficiles à maitriser;
- Ces perturbations peuvent retentir sur le rendement au travail;
- Pas d’études tunisiennes publiées dans ce contexte.

### Contribution de notre étude à la connaissance

- Souligner et quantifier la détresse psychologique du personnel d’anesthésie réanimation;
- Mettre l’accent sur l’importance de l’identification des facteurs générateurs de détresse psychologique afin de proposer des solutions adaptées et améliorer le rendement au travail.

## Conflits d’intérêts

Les auteurs ne déclarent aucun conflit d’intérêts.

## References

[1] Lesage F-X, Berjot S, Amoura C, Deschamps F, Grebot E (2012). Mesure du stress en milieu de travail par autoquestionnaires validés en français: revue de la littérature. Archives des Maladies Professionnelles et de l'Environnement.

[2] Ponchet M, Geeraerts T (2011). Le stress de l’anesthésiste-réanimateur.

[3] Karasek RA (1979). Job demands, job decision latitude and mental strain: implications for job redesign. Adm Sci Q.

[4] Hamilton M (1959). The assessment of anxiety states by rating. Br J Med Psychol.

[5] Siegrist J (1996). Adverse health effects of high effort - low reward conditions at work. J Occup Health Psychol.

[6] Mager Stellman J (2000). Encyclopédie de sécurité et de santé au travail-Volume.

[7] Kain ZN, Chan KM, Kaz JD, Fleisher L, Doler J, Rosenfeld LE (2002). Anesthesiologists and acute perioperative stress: a cohort study. Anesth Analg.

[8] Malmberg B, Persson R, Jonsson BA, Erfurth EM, Flisberg P, Ranklev E, Orbaek P (2007). Physiological restitution after night-call duty in anaesthesiologists: impact on metabolic factors. Acta Anaesthesiol Scand.

[9] Embriaco N, Azoulay E, Barrau K, Kentish N, Pochard F, Loundou A, Papazian L (2007). High level of burnout in intensivists: prevalence and associated factors. Am J Respir Crit Care Med.

[10] Poncet MC, Toullic P, Papazian L, Kentish-Barnes N, Timsit JF, Pochard F, Chevret S, Schlemmer B, Azoulay E (2007). Burnout syndrome in critical care nursing staff. Am J Respir Crit Care Med.

[11] Massou S, Doghmi N, Belhaj A, Aboulaala K, Azendour H, Haimeur C, Balkhi H, Drissi Kamili N (2013). Enquête sur le syndrome d’épuisement professionnel chez les personnels d’anesthésie réanimation de quatre hôpitaux universitaires marocains. Ann Med Psychol.

[12] Magalhães E, Oliveira ÁC, Govêia CS, Ladeira LC, Queiroz DM, Vieira CV (2015). Prevalence of burnout syndrome among anesthesiologists in the Federal District. Rev Bras Anestesiol.

[13] Laurent A, Chahraoui K (2012). L’impact du stress professionnel sur les intervenants SMUR. Pratiques Psychologiques.

[14] Larsson J, Rosenqvist U, Holmstrom I (2007). Enjoying work or burdened by it? How anaesthesists experience and handle difficulties at work: a qualitative study. Br J Anaesth.

[15] Sobrequés J, Cebrià J, Segura J, Rodríguez C, García M, Juncosa S (2003). Job satisfaction and burn out in general practitioners. Aten Primaria.

[16] Truchot D (2008). Career orientation and burn out in French general practitioners. Psychol Rep.

[17] Garrosa E, Moreno-Jiménez B, Liang Y, Gonzalez JL (2008). The relationship between sociodemographic variables, job stressors, burn out and hardy personality in nurses. Int J Nurs Stud.

[18] Fahrenkopf AM, Sectish TC, Barger LK, Sharek PJ, Lewin D, Chiang VW, Edwards S, Wiedermann BL, Landrigan CP (2008). Rates of medication errors among depressed and burnt out residents: prospective cohort study. BMJ.

[19] Howard SK, Rosekind MR, Katz JD, Berry AJ (2002). Fatigue in anesthesia: implications and strategies for patient and provider safety. Anesthesiology.

[20] Businger A, Guller U, Oertli D (2010). Effect of the 50-hour workweek limitation on training of surgical residents in Switzerland. Arch Surg.

[21] Kinzl JF, Traweger C, Trefalt E, Riccabona U, Lederer W (2007). Work stress and gender-dependent coping strategies in anesthesiologists at a university hospital. J Clin Anesth.

[22] Servant D (2012). Gestion du stress et de l'anxiété-3e édition.

[23] Endler NS (1997). Stress, anxiety and coping: the multidimensional interaction model. Canadian Psychology.

